# Identification of pyroptosis related subtypes and tumor microenvironment infiltration characteristics in breast cancer

**DOI:** 10.1038/s41598-022-14897-1

**Published:** 2022-06-23

**Authors:** Guo Huang, Jun Zhou, Juan Chen, Guowen Liu

**Affiliations:** 1 Hengyang Medical School, University of South China, Hengyang, 421001 Hunan China; 2 Key Laboratory of Tumor Cellular and Molecular Pathology, College of Hunan Province, Cancer Research Institute, University of South China, Hengyang, 421001 Hunan China; 3 The Second Affiliated Hospital, Department of Radiotherapy, Hengyang Medical School, University of South China, Hengyang, 421001 Hunan China; 4 Department of Thyroid and Breast Surgery, The First Affiliated Hospital of Shenzhen University, Shenzhen Second People’s Hospital, Shenzhen, 518035 Guangdong China

**Keywords:** Breast cancer, Immunology, Oncology, Risk factors

## Abstract

Understanding the association of pyroptosis with tumor progression, prognosis and effect on immunotherapeutic response in breast cancer (BC) is limited. This study analysed forty pyroptosis-related genes to construct the pyroptosis score. Association of the pyroptosis score with the overall survival, clinical features, tumor mutation load, immune cell infiltration, and treatment sensitivity of patients with BC was analysed. Out of 983 BC samples, 304 (30.93%) had genetic alterations with the highest TP53 frequency. We identified three separate subtypes associated with pyroptosis action. These subtypes correlate with the clinicopathological characteristics, TME immune cell infiltration, and disease prognosis. Based on the expression levels of the pyroptosis genes, we divided the pyroptosis score into a high group and a low group. The immune-activated pyroptosis subtype had a higher score with a better prognosis. We also observed that the pyroptosis score correlates with the tumor mutation burden. The pyroptosis score and disease prognosis were directly proportional. A higher pyroptosis score indicated a better prognosis. Results suggest that the pyroptosis-related gene prognosis model is closely related to the immune cell infiltration of BC. The three pyroptosis subtypes associated with BC assist in accurately identifying the tumor subtype, the prognosis of immunotherapy drugs and the patient’s therapeutic response.

## Introduction

Breast cancer has become the most prevalent type of cancer in females globally by crossing the ratio of lung cancer, with approximately 2 million 300 thousand new cases. It has also become the fifth leading cause of mortalities due to malignancies, with 685,000 reported deaths^1^. Currently, there are four subtypes of BC based on the variable expression levels of PR, ER, Her-2, and Ki67. The most common subtype is the hormone receptor-positive breast cancer (HR+) which accounts for about 70% of the global breast cancer occurrence^2^. Presently, the major treatment options for breast cancer include surgery^3^, chemotherapy (neoadjuvant chemotherapy and postoperative adjuvant chemotherapy)^4^, radiotherapy^5^, endocrine therapy^6^, anti-HER-2 therapy^7^, and immunotherapy^8^. Breast cancer accounts for around 30% of all cancer deaths in women, the patients with breast cancer do not primarily die from cancer, rather, resistance to chemotherapeutic drugs, metastasis, and recurrence are the leading causes of the increased mortality related to it. Once cancer spreads as a result of metastasis, the 5-years survival rate is only 25%^9^.

Pyroptosis is a type of programmed cell death that includes the formation of cell pores mediated by the Gasdermin protein family, causing cell swelling and eventual rupture due to which inflammatory mediators such as interleukin (IL)-1β, IL-18, HMGB1 are released thereby promoting an inflammatory response^10^. The pyroptosis activation pathway is sorted into the classical cell pyroptosis pathway in which caspase-1 is activated by the inflammatory bodies and the non-classical cell pyroptosis pathway in which cytoplasmic lipopolysaccharide (LPS) activates caspase-4/5/11^11^. Pyroptosis is not only known for its significant role in inflammatory diseases such as acute severe pancreatitis^12^, lupus nephritis^13^, gastrointestinal inflammation^14^, sepsis^15^, but it also plays a substantial role in the inhibition of tumors or drug targeting in malignant tumors like lung cancer^16^, malignant melanoma^17^, liver cancer^18^, and esophageal squamous cell carcinoma^19^. The correlation between pyroptosis and the immune system depends on complex cellular interactions, including pyroptosis and immune cells^20^. The pyroptosis of normal cells has the ability to alter the tumor microenvironment (TME) and accelerate immune escape^21^. Meanwhile, there are therapeutic techniques that engage the immune system and cause the pyroptosis of cancer cells^22^. The pyroptosis in TME produces inflammatory bodies and cytokines that play a potential tumor-promoting role^23^. The altered and abnormal expression of the regulatory genes involved in pyroptosis is closely correlated with immune regulation and the progression of malignant tumors.

Previous studies indicate that breast cancer has poor immunogenicity. However, with the development in research, the immunological features of breast cancer have caught further attention^24^. Initial studies have shown that breast cancer has a high tumor mutation load (TMB)^25^. Later, it was reported that some breast cancer patients had higher expression of programmed cell death ligand-1 (PD-L1). In Triple negative breast cancer (TNBC), the positive rate of PD-L1 was about 20%^26^, and the expression of PD-L1 was positively correlated with the density of tumor-infiltrating lymphocytes (TILs)^27^. Tumor immunotherapy works by killing the tumor cells through the reactivation of the body's anti-tumor immune response. The research in the area of breast cancer immunotherapy mainly focuses on chimeric Antigen Receptor T-Cell Immunotherapy (CAR-T), vaccines, and immunosuppressive point inhibitors (ICIs). In breast cancer, the Gasdermin E (GSDME) expression level has a close correlation to the ER status. Oftentimes, hypermethylation of the GSDME gene is noticed in the ER-positive breast cancer cells which are positively correlated with lymph node metastasis of the BC^28^. The objective of this study is to explore the subtypes of BC based on the regulatory genes related to pyroptosis, construct a pyroptosis scoring system and prognosis model, evaluate the differences of immune cell infiltration, and deeply understand the characteristics of TME cell infiltration controlled by various pyroptosis genes. This could aid in better learning of the mechanism of BC carcinogenesis as well as the prediction of immunotherapy response.

## Materials and methods

### Data sources

The RNA sequencing (RNA-Seq) and tumor mutation burden (TMB) data of 1098 breast cancer patients were collected along with 113 normal breast samples from The Cancer Genome Atlas (TCGA, https://portal.gdc.cancer.gov). The IPS of CTLA-4 and PD-1 blockers in BC were downloaded from the TCIA database. The website of the University of California, Santa Cruz (UCSC) was used to obtain copy number variation (CNV) data. The GSE20685 (contains 327 breast cancer cases)^29^, GSE88770(contains 117 breast cancer cases)^30^, GSE41119(contains 287 breast cancer cases)^31^ and GSE42568(contains 104 breast cancer cases and 17 normal breast cases)^32^ breast cancer dataset was taken from the Gene Expression Omnibus (GEO, https://www.ncbi.nlm.nih.gov/geo/). The ComBat function normalized all the RNA-Seq data. Forty pyroptosis genes related to BC were provided by GSEA. The CNV data, differential expression, mutation, gene type, and correlation of pyroptosis-related genes (PRGs) in breast cancer were analyzed. All analyses were performed according to the publication guidelines provided by TCGA and GEO databases. From this dataset, we obtained a total of 40 pyroptosis-related genes (Table 1) using a molecular signature database (http://www.gsea-msigdb.org/gsea/), and involved the Pyroptosis (M41805) and prior review^33^.

**Table 1:** Pyroptosis gene members.

Gene	Gene description
AIM2	Absent in melanoma 2
APIP	APAF1 interacting protein
BAK1	BCL2 antagonist/killer 1
BAX	BCL2 associated X
CASP1	Caspase 1
CASP3	Caspase 3
CASP4	Caspase 4
CASP5	Caspase 5
CASP8	Caspase 8
CHMP2A	Charged multivesicular body protein 2A
CHMP2B	Charged multivesicular body protein 2B
CHMP3	Charged multivesicular body protein 3
CHMP4A	Charged multivesicular body protein 4A
CHMP4B	Charged multivesicular body protein 4B
CHMP4C	Charged multivesicular body protein 4C
CHMP6	Charged multivesicular body protein 6
CHMP7	Charged multivesicular body protein 7
CYCS	Cytochrome c, somatic
DHX9	DEAH (Asp–Glu–Ala–His) box helicase 9
ELANE	Elastase, neutrophil expressed
GSDMA	Gasdermin A
GSDMB	Gasdermin B
GSDMC	Gasdermin C
GSDMD	Gasdermin D
GSDME	Gasdermin E
GZMA	Granzyme A
GZMB	Granzyme B
HMGB1	High mobility group box 1
IL18	Interleukin 18
IL1A	Interleukin 1A
IL1B	Interleukin 1B
IRF1	Interferon regulatory factor 1
IRF2	Interferon regulatory factor 2
NAIP	NLR family, apoptosis inhibitory protein
NLRC4	NLR family, pyrin domain containing 4
NLRP1	NLR family, pyrin domain containing 1
NLRP9	NLR family, pyrin domain containing 9
TP53	Tumor protein p53
TP63	Tumor protein p63
ZBP1	Z-DNA binding protein 1

### Survival analysis

Univariate Cox regression analysis was used to analyze the prognostic method and P < 0.05 was regarded as statistically significant. Correlation of pyroptosis genes to BC (Table 2). The survival curve was plotted by the Kaplan–Meier.

**Table 2:** Univariate analysis showing associations between pyroptosis-related gene in BRCA.

Gene	HR	HR.95L	HR.95H	P value	km
AIM2	0.912715445	0.813325743	1.024250728	0.120513297	0.009392316
APIP	1.016592982	0.81207352	1.272620353	0.885819254	0.257143795
BAK1	1.162944481	0.932377649	1.450527978	0.180593541	0.02819334
BAX	1.121129801	0.890461544	1.411551165	0.330632098	0.094350569
CASP1	0.884210567	0.771020013	1.014018201	0.078275613	0.009589514
CASP3	1.09666963	0.861601996	1.395869883	0.453425357	0.085492897
CASP4	0.854109359	0.704929456	1.034859292	0.10737451	0.002572295
CASP5	0.995666924	0.811753723	1.221248016	0.966756093	0.229638302
CASP8	0.813628256	0.649094287	1.019868688	0.073568466	0.016752102
CHMP2A	0.909284864	0.738084579	1.120195419	0.371583564	0.009311718
CHMP2B	1.329704534	1.053905967	1.677677328	0.016276659	0.007690403
CHMP4B	0.895934772	0.68591719	1.170256595	0.420060156	0.04414922
CHMP4C	1.217934201	1.056302216	1.404298595	0.006648363	0.000769898
CHMP6	0.918498603	0.736011624	1.146231467	0.451881956	0.003893996
CHMP7	0.733102856	0.584389174	0.919660769	0.007275175	8.65E−05
CYCS	1.285578007	1.032526435	1.600647457	0.024693266	0.000723235
DHX9	1.099189157	0.881927293	1.369973253	0.39995239	0.028986248
ELANE	0.805921553	0.654121728	0.992949052	0.042721933	0.017983067
GSDMB	1.091277291	0.983432398	1.210948642	0.099910879	0.029756998
GSDMC	1.184343722	1.078298218	1.300818296	0.000407705	0.000122464
GSDMD	0.873262217	0.750100238	1.016646659	0.080616297	0.000246088
GZMA	0.837548491	0.766268389	0.915459237	9.37E−05	2.35E−05
GZMB	0.883817681	0.815107897	0.958319378	0.002780453	0.00083888
HMGB1	1.027082635	0.782357247	1.348359388	0.847399694	0.269646314
IL18	0.835880945	0.733167673	0.952983852	0.007365334	0.001176789
IL1A	1.025641783	0.789992786	1.331583129	0.84923728	0.016975195
IL1B	0.894226392	0.780204863	1.024911377	0.108186323	0.034913794
IRF1	0.764678357	0.656387974	0.890834403	0.000573894	0.000366415
IRF2	0.701467775	0.546188904	0.90089168	0.005477467	0.001206426
NAIP	0.762566602	0.571231818	1.017989202	0.065914651	0.015491051
NLRC4	1.11137458	0.912476715	1.353627373	0.293910104	0.018471531
NLRP1	0.86880861	0.730541062	1.033245686	0.111798422	0.01879444
NLRP9	1.065105384	0.855783846	1.325626189	0.572092589	0.202949253
TP53	1.001618075	0.860236442	1.166236072	0.983385383	0.058196864
TP63	0.879544184	0.796418963	0.971345496	0.01127922	1.30E−05
ZBP1	0.910981567	0.813507511	1.020134913	0.106373757	0.001694627

Unadjusted HRs are shown with 95% confidence intervals.

### Consistency cluster analysis and differential gene analysis

Consensus clustering software package (https://bioconductor.org/) was used for clustering and typing on the basis of the co-expression of 40 pyroptosis regulatory genes. We used the “sva” package to merge GSE88770, GSE41119 and GSE42568 GEO datasets as a validation dataset, then analyzed and validated our pyroptosis subtypes. A single sample gene set enrichment analysis (ssGSEA) algorithm helped in measuring the immune cells (28 kinds of immune cells) content following a comparison of the infiltration of immune cells among different types of pyroptosis^34^. Differentially expressed genes (DEGs) were obtained from the TCGA dataset of the GEO dataset. Subsequently, univariate Cox analysis was performed, P < 0.05 was filtered to obtain DEGs related to patients’ prognosis, and consistent cluster classification was carried out according to the prognostic DEGs to the pyroptosis subtype based on DEGs. Gene Ontology (GO) and Kyoto Encyclopedia of Genes and Genomes^35^ (KEGG) were used for the analysis of DEGs.

### Scoring construction and analysis of immune-related indexes of pyroptosis

Principal component analysis^36^ (PCA) was used for the evaluation of the prognostic DEGs to attain a P-score (Pyroptosis-score = PCA1 + PCA2). The relationship between P-score and immune cells, TMB, different clinical features (Gender, Age, Stage, T, N, M), and PD-L1 was analyzed.

### Construction of pyroptosis related prognosis model

We performed a single factor Cox regression analysis on DEG. All BC patients were randomly classified in a ratio of 1:1 into two separate groups called the training group (n = 708) and a test group (n = 708). Furthermore, we used the Lasso Cox regression algorithm, the “glmnet” R package helped in minimizing the risk of overfitting, and the risk prediction model was established by 10× cross-validation^37,38^. Multivariate Cox analysis helped in the selection of candidate genes (Supplemental Table S1) and also in the establishment of a risk score (risk score) = Σ (EXPI × coefi)), coefi while the EXPI represents the risk coefficient and expression of each respective gene. The 708 patients in the training group were split into two groups based on their median risk score: a low-risk group and a high-risk group. We then ran the Kaplan–Meier survival analysis and derived a receiver operating characteristic curve.

### Comparison of pyroptosis-related gene Signature with other breast cancer pyroptosis models

To determine whether our 16 genes associated with pyroptosis genes are superior to other breast cancer pyroptosis models, we used the subject work curve (ROC) to compare 17-gene Signature^39^, 15-gene Signature^40^, and 3 gene Signature^41^. The 1, 3, and 5-year ROC curves constructed for the all TCGA cohort were compared with 16 genes Signature associated with scorch death in this study to assess the advantages and disadvantages of each model. Compare C-index and RMS at the same time.

### Evaluate the immune status between high and low-risk groups

CIBERSORT^42^ was used to measure the quantity of 22 invasive immune cells in heterogeneous samples of the low and high-risk groups in order to estimate the ratio of TICs in TME. The correlation between the P-score and 22 types of infiltrating immune cells, as well as the association between TMB and P-score was investigated in this study.

### Analysis of gene mutation and drug sensitivity

The “maftools” R package was used to build the mutation annotation format (MAF) in the TCGA database for observing somatic mutations in BC patients between the high and low-risk categories^43^. In both groups, the TMB score was also measured for each BC patient. To observe the difference in the efficacy of chemotherapeutic drugs between the two groups, the “pRRophetic” package was used to calculate the half maximal inhibitory concentration (IC50) of these drugs that are widely used for treating BC.

### Establishment and verification of nomograph scoring system

Using the “rms” software tool, the clinical traits and risk scores were combined to build a prediction nomogram^44^. A score was assigned to each variable in the nomograph scoring system, and then added the scores of all variables for each sample to get the total score. For 1, 3, and 5-year survival, time-dependent ROC curves were used to evaluate Nomograms. The values between the anticipated 1, 3, and 5-year survival events and the observed results were described using the nomogram calibration plot.

### Ethical approval

As this work is a bioinformatics analysis, ethical approval is not required. All methods were performed in accordance with the relevant guidelines and regulations.

### Statistical analysis

We normalized all RNA-Seq data by the ComBat function in the sva software package. Wilcoxon rank sum test was performed to check the difference of gene expression between normal tissues and tumor tissues. The survival curve was drawn with the help of the Kaplan–Meier method, clustering classification was carried out by consensus clustering software package, and the ssGSEA algorithm helped in the evaluation of tumor-infiltrating immune cells. All statistics were completed using the R language software package (https://www.r-project). We considered P-value < 0.05 as significant.

### Consent for publication

All author knows the situation and agrees to publish.

## Results

### Variation and prognosis of pyroptosis regulatory genes in BC

**Figure 1:**
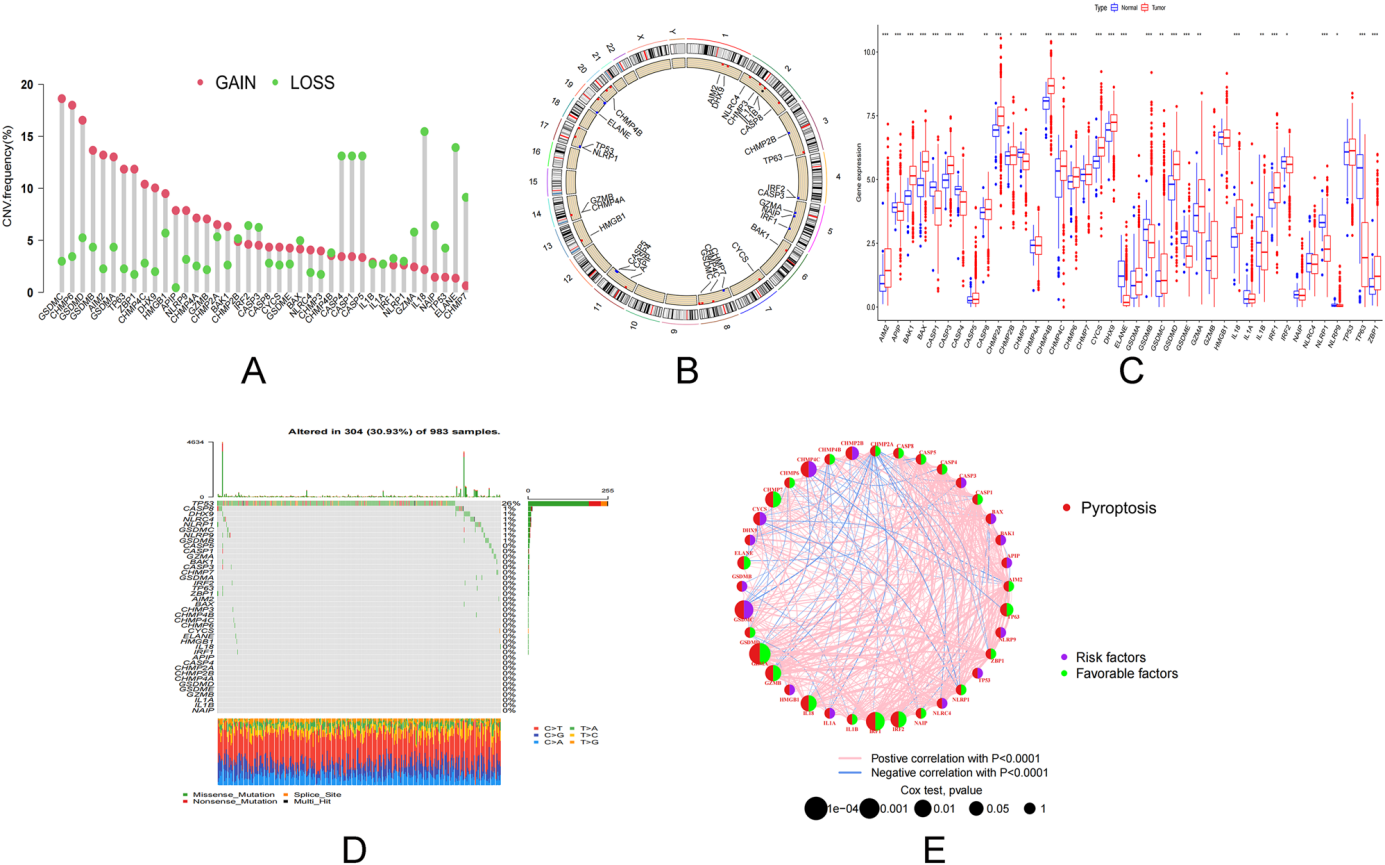
Transcriptional and genetic alterations of PRGs in BC. (**A**) Frequency of CNV gain, loss and non-CNV with PRGs. (**B**) Location of CNV alterations in PRGs on 23 chromosomes. (**C**) Distribution of 40 PRGs expression in normal and BC tissues. (**D**) Frequency of mutations in 40 PRGs in 1098 BC samples from the TCGA cohort. (**E**) Interaction between PRGs in BC. The lines connecting the PRGs represent their interactions and the thickness of the lines indicates the strength of the association between the PRGs. Green and pink represent negative and pink-positive associations, respectively. *PRGs* pyroptosis-associated genes, *BC* breast cancer, *TCGA* cancer genome atlas, *CNV* copy number variant.

Initially, we studied the mutation frequency of CNV, insertion, and deletion of copy number of 40 pyroptosis regulatory genes found in BC (Fig. 1A,B). At the same time, researchers looked at the expression of 40 pyroptosis genes in malignancies and normal tissues. CASP5, CHMP4A, CHMP7, GSDMA, HMGB1, IL1A, NAIP, NLRC4 and TP53 expressions were determined to be the same (Fig. 1C). Changes in pyroptosis regulating genes were detected in 304 of 983 BC patients, with a frequency of 30.93%. Missense mutations, splice-site mutations, and nonsense mutations are the most common types of mutations. The most frequently mutated gene was TP53, which was followed by CASP8 and DHX9 (Fig. 1D). To learn more about how the pyroptosis regulatory genes interact, we developed a network diagram of survival and interaction between pyroptosis regulatory genes (Fig. 1E). Survival analysis revealed that 29 PRGs were closely associated with prognosis. Patients with high expression of AIM2, CASP1, CASP4, CASP8, CHMP2A, CHMP4B, CHMP6, CHMP7, ELANE, GSDMD, GZMA, GZMB, IL1A, IL1B, IL18, IRF1, IRF2, NAIP, NLRC1, TP63 and ZBP1 had a better prognosis, while patients with low expression of these genes had a better prognosis. Patients with low expression of BAK1, CHMP2B, CHMP4C, CYCS, DHX9, GSDMB, GSDMC and NLRC4 had a better prognosis (Supplementary Fig. S1). These findings reveal that the expression of pyroptosis regulating genes differ significantly between normal and malignant tissues. Simultaneously, it has been established that pyroptosis regulating genes influence the prognosis of BC patients.

### Pyroptosis subtypes and gene subtypes based on regulatory genes

**Figure 2:**
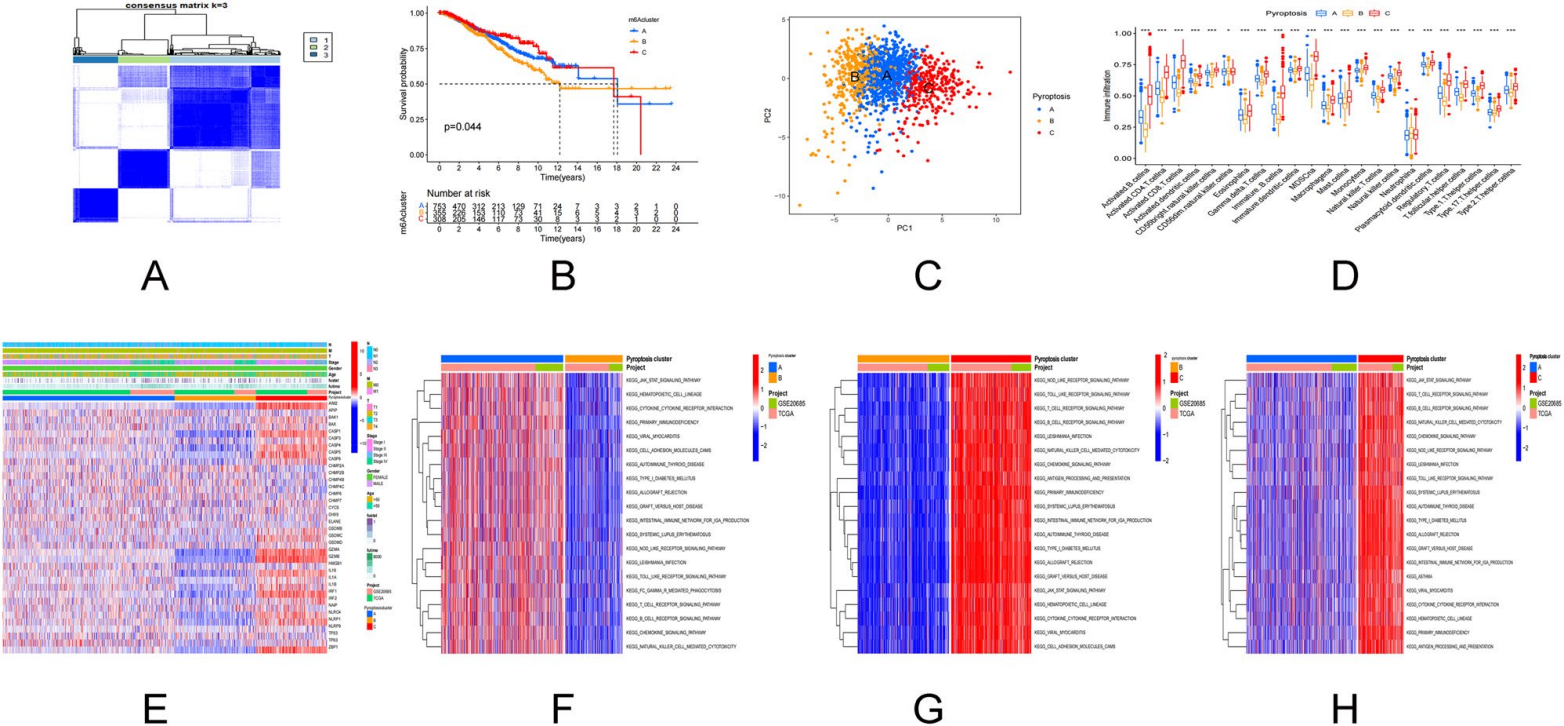
PRGs subtypes and clinicopathological features. (**A**) Consensus matrix heat map defining the three clusters (k = 3) and their associated regions. (**B**) Survival analysis of the three subtypes versus OS. (**C**) PCA analysis showing significant differences in transcriptomes between the three subtypes. (**D**) Association of the three subtypes with immune cells infiltration. (**E**) Differences in the three subtypes in relation to clinicopathological features and expression levels of PRG. (**F–H**) GSVA of biological pathways between the three different isoforms, where red and blue represent activating pathways and blue represent inhibiting pathways, respectively. *PCA* principal component analysis, *OS* overall survival.

**Figure 3:**
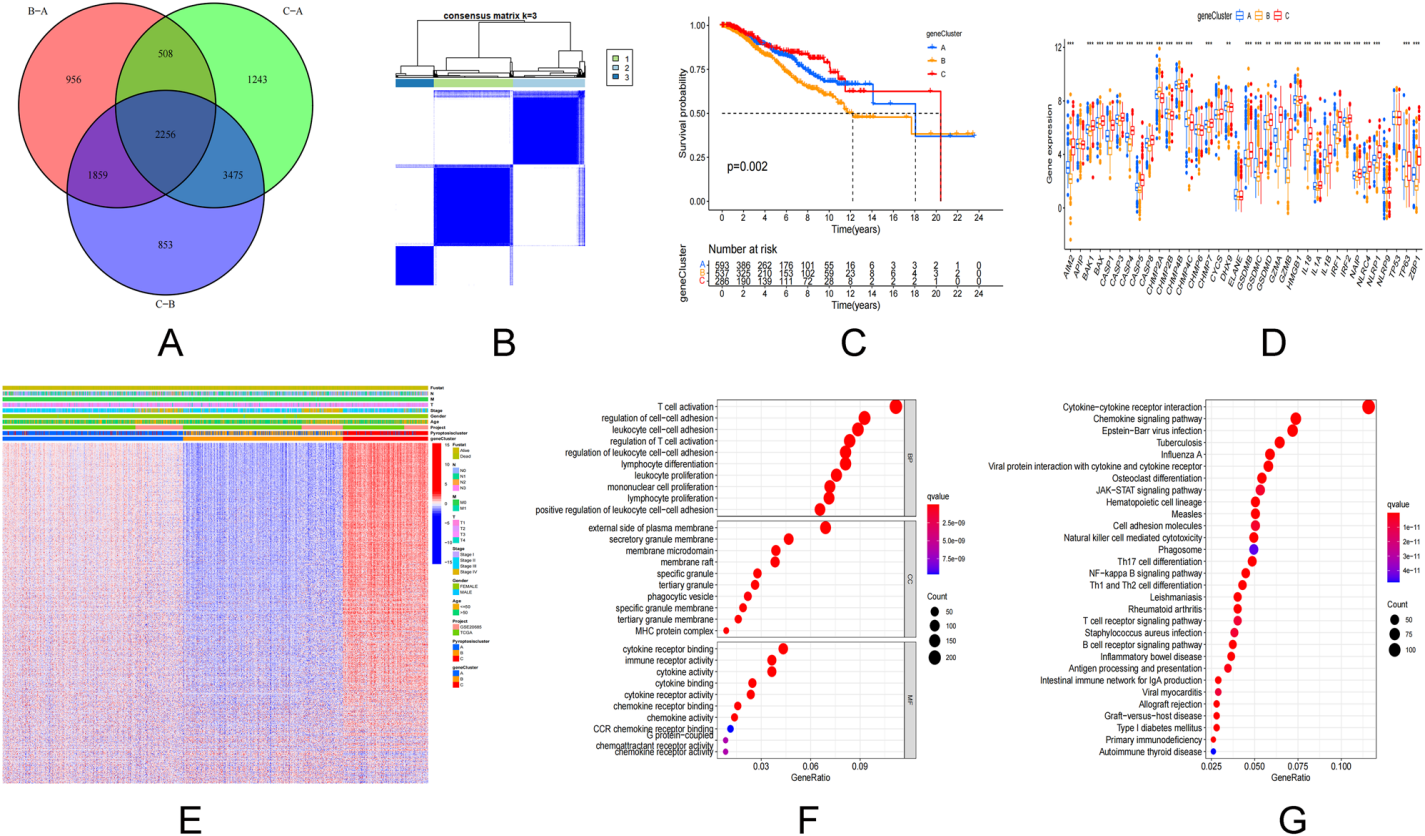
Gene subtypes based on PRGs. (**A**) Venn diagram showing DEGs for the three pyroptosis subtypes. (**B**) Consensus matrix heat map defining the three genetic subtypes subtypes (k = 3). (**C**) Survival analysis of the three genetic subtypes versus overall survival. (**D**) Expression distribution of the 40 PRGs in the three genetic subtypes. (**E**) Differences in expression levels of the three genetic subtypes in relation to clinicopathological features and PRGs. (**F,G**) GO and KEGG enrichment analysis of DEGs in the three genetic subtypes.

The TCGA-BC data set and GSE20685 data set were clustered on the basis of 40 pyroptosis regulatory genes. It is categorized into three pyroptosis subtypes based on the cumulative distribution function (CDF) curve and area under the curve (AUC) of consensus score (Fig. 2A). The three pyroptosis regulating gene cluster groupings demonstrated statistical differences in survival (Fig. 2B). The major components of pyroptosis regulating genes were evaluated using the PCA. Three pyroptosis subtypes were discovered to be easily distinguishable (Fig. 2C). Similar results can be obtained for our validation data (Supplementary Fig. S2A–D). We next used ssGSEA to look at the number of immune cells infiltration in BC tumor samples and examined the difference between the three pyroptosis subtypes. It was observed that all three pyroptosis subtypes had a considerable number of immune cells and a high level of infiltration (Fig. 2D). The link between the three pyroptosis subtypes, pyroptosis regulatory genes, and clinicopathological parameters was then investigated. The survival rate of stage I patients increased dramatically in the pyroptosis cluster C age ≤ 50 years, and the expression of the pyroptosis gene increased substantially (Fig. 2E). Pyroptosis cluster C demonstrated enrichment pathways related to immune activation, including T cell receptor signal pathway, B cell receptor signal pathway, nod like receptor signal pathway, toll-like receptor signal pathway, chemokine signal pathway, cytokine receptor interaction, and JAK/STAT signal pathway according to GSVA enrichment analysis based on the three subtypes of pyroptosis regulatory genes (Fig. 2G,H). Pyroptosis cluster B was greatly linked with immunosuppression (Fig. 2F). We identified pyroptosis cluster C as an immunoinflammatory phenotype defined by adaptive immune cell infiltration and immunological activation based on the results of the aforementioned analysis. Pyroptosis cluster B is classified as an immunosuppressive phenotype known for its immunosuppression. Pyroptosis cluster A serves as a link between Pyroptosis cluster C and B. After that, gene analysis was carried out within the pyroptosis cluster groups, 2256 DEGs obtained by univariate Cox analysis (Fig. 3A). The consistency clustering was repeated, and three gene clustering types were identified as gene cluster A, B, and C (Fig. 3B). The results of survival analysis revealed a significant variation in prognosis (P = 0.002, Fig. 3C). At the same time, researchers analyzed the expression of 40 pyroptosis genes in tumors and normal tissues. The expression of the remaining 35 genes was different, except for APIP, CHMP6, CYCS, ELANE and TP53, which had no difference in expression (Fig. 3D). DEG-based analysis revealed that these subgroups had distinct clinicopathological features. Gene cluster A has a favorable prognosis for T1, N0, and stage I cancers (Fig. 3E).

### Enrichment function of DEGs and clinical correlation analysis

Through GO enrichment analysis of DEGs, the top 5 biological processes containing T cell activation, regulation of cell–cell adhesion, leucocyte cell–cell adhesion, regulation of T cell activation, regulation of leucocyte cell–cell adhesion. The top five cell components containing external side of the plasma membrane, secret granular membrane, membrane microdomain, membrane raft, and specific granular. And the molecular functions which include cytokine receptor binding, immune receptor activity, cytokine activity, cytokine receptor activity, and cytokine binding (Fig. 3F). The cytokine–cytokine receptor interaction pathway, chemokine signaling pathway, Epstein–Barr virus infection pathway were considerably enriched in KEGG pathway analysis (Fig. 3G).

### Construction of pyroptosis score and its clinical significance

**Figure 4:**
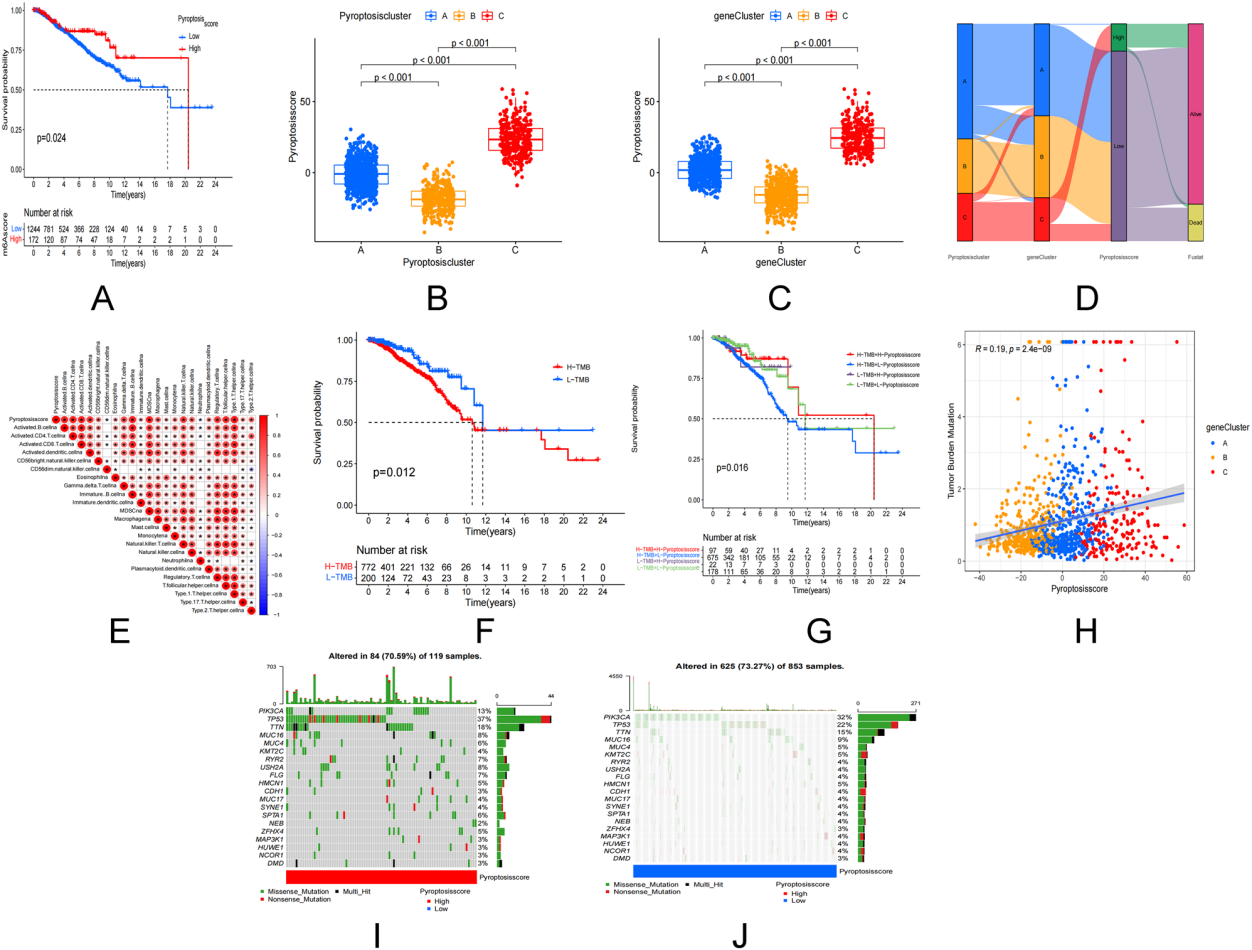
Construction of the pyroptosis score. (**A**) Prognostic analysis of the pyroptosis score. (**B**) Relationship between pyroptosis score and pyroptosis subtypes. (**C**) Relationship between pyroptosis score and genotyping. (**D**) Sankey plots of different pyroptosis subtypes (**A–C**), pyroptosis genetic clustering subtypes and pyroptosis score in relation to overall survival status. (**E**) Correlation analysis of pyroptosis score with 28 immune cell types. (**F**) Prognostic analysis of TMB. (**G,H**) Prognosis and correlation between pyroptosis score and tumor mutational load. (**I,J**) Waterfall plot of somatic mutation characteristics in high and low pyroptosis scores. Each column represents an individual patient. The upper bar graph shows TMB and the numbers on the right indicate the frequency of mutations in each gene. The bar on the right shows the proportion of each mutation type.

The pyroptosis regulatory gene has been discovered to have a regulatory effect on the breast cancer prognosis, cytokines, and immune infiltration of breast cancer. These conclusions, however, are predicted by the BC results. Presently, they cannot predict the pattern of pyroptosis regulatory genes accurately in a single BC patient. As a result, pyroptosis-score (P-score = PCA1 + PCA2) was used to quantify the pattern of pyroptosis regulatory genes in individual BC patients, and predict the patient’s treatment response and prognosis using PCA based on DEGs. The pyroptosis subtype was linked to the gene subtype’s pyroptosis score, with individuals having a higher score having a better prognosis (P = 0.024, Fig. 4A). A positive prognosis was indicated by Cluster A and Cluster C as well as higher pyroptosis scores (Fig. 4B–D). Simultaneously, the chosen DEGs regulate T cell activation and regulation, as well as cytokines and chemokines, and are closely linked with the clinicopathological characteristics. Activated CD4+ T cells, CD8+ T cells, B cells, dendritic cells, natural killer T cells, regulatory T cells, T follicular Helper cell, and type 1 are the immune cells among which the pyroptosis score was favorably associated with T helper cells (Fig. 4E). TMB is closely linked to a patient’s prognosis. The prognosis of low and medium TMB in BC patients is better according to this study (Fig. 4F). Patients with a high pyroptosis score, even if they have a high TMB, have a better overall prognosis (Fig. 4G). TMB, genotyping, and pyroptosis scores are all positively linked (R = 0.19, Fig. 4H). 84 of 119 BC patients with high pyroptosis scores had gene mutations with a frequency of 70.59% (Fig. 4I). 625 of 853 BC patients with low pyroptosis scores had gene mutations with a frequency of 73.27% (Fig. 4J). A high pyroptosis score is a consistent predictor of outcome in BC patients under the age of 50 who are female and have no lymph node metastasis (Supplementary Fig. S3A). On the other side, we discovered a link between pyroptosis score and age, gender, lymph node metastasis, stage, and size of the tumor (Supplementary Fig. S3B).

### Comparison of genetic features associated with other models of prognosis of pyroptosis in breast cancer

To determine whether our 16 pyroptosis related genes model were superior to other breast cancer pyroptosis models, by comparing them with the 17-gene model, the 15-gene model, and the 3 gene model, our pyroptosis model AUC values at 1, 3, and 5 years that were statistically significant (Supplemental Fig. S4A–D), The OS of the pyroptosis model was s statistically significant (SupplementalFig. S4E–H). Our C-index and RMS index (Supplemental Fig. S4G–I) were higher than those of the other three models. Indicate that our model predicts well.

### Immunotherapy

**Figure 5:**
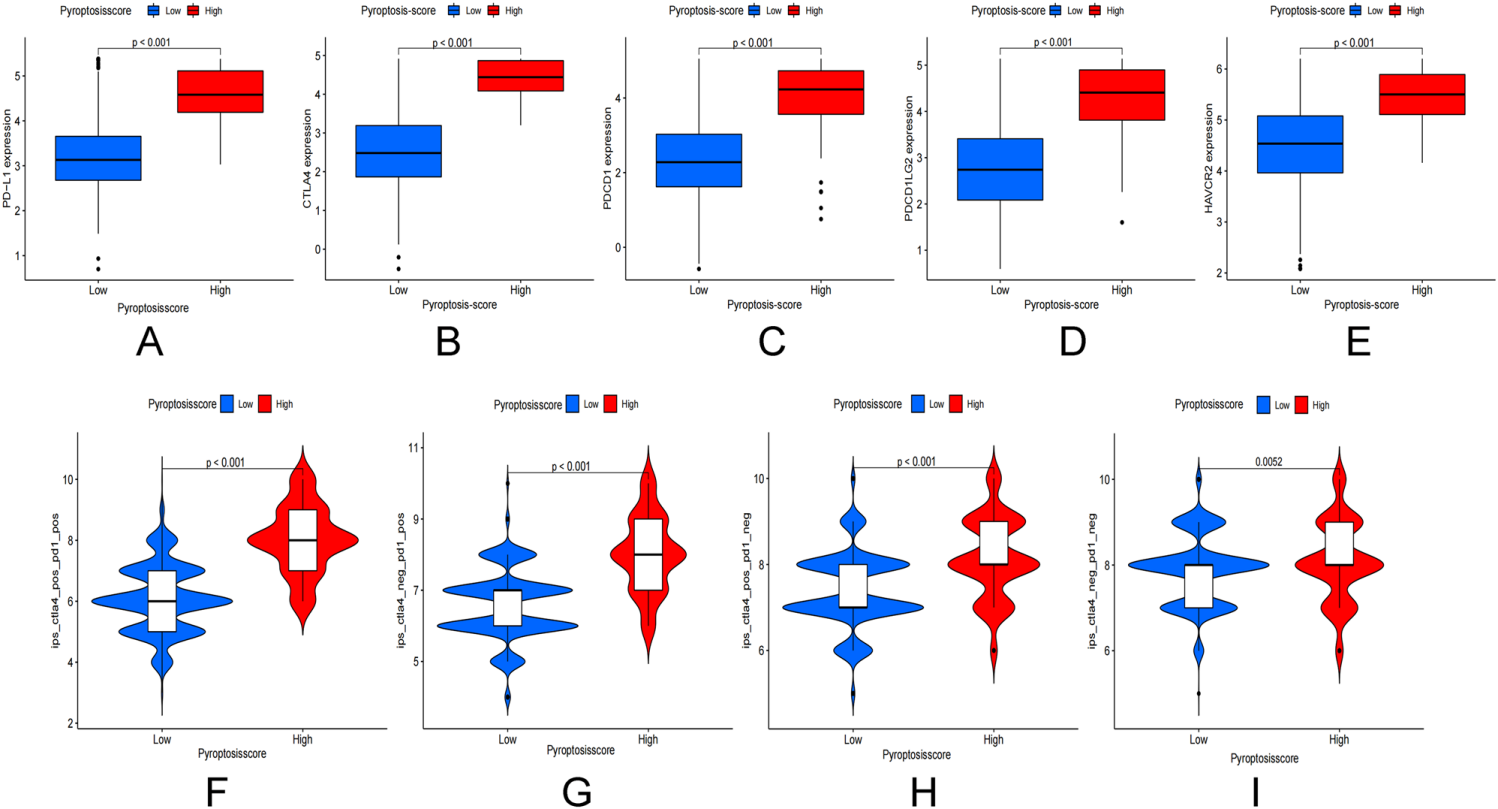
Correlation between pyroptosis score and immune blocking sites. (**A–E**) Correlation analysis between PD-L1, CTLA4, PDCD1, PDCD1LG2, HAVCR2 expression and pyroptosis score. (**F–I**) Correlation between IPS immunotherapy and pyroptosis score.

Breast cancer immunotherapy now focuses primarily on PD-L1 and CTLA4. The expression of PD-L1, CTLA4, PDCD1, PDCD1LG2, and HAVCR2 was found to be significantly higher in the high pyroptosis score group (Fig. 5A–E). We discovered that when CTLA4 was positive or PD-L1 was positive, and both CTLA4 and PD-L1 were positive, the immunotherapy scores were higher than in patients with negative CTLA4 and PD-L1 (Fig. 5F–I).

### Construction of prognosis prediction model of pyroptosis gene

**Figure 6:**
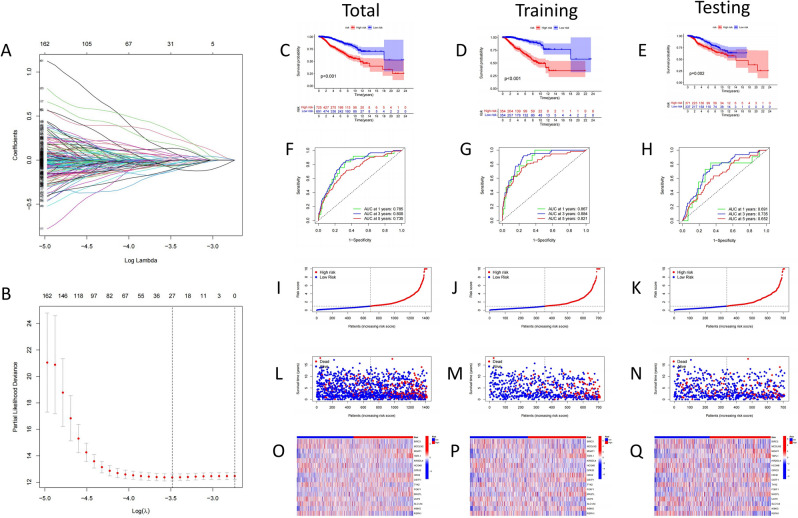
Predictive model for pyroptosis-associated genes. (**A,B**) LASSO Cox regression analysis of 16 pyroptosis-associated genes. (**C–E**) Overall survival analysis of high/low risk patients. (**F–H**) Time-dependent ROC analysis of risk scores in predicting survival. (**I–K**) Risk scores for each patient in the total cohort, training group, and validation group. (**L–N**) Number of deaths in the high- and low-risk groups in the cohort. (**O–Q**) Expression patterns of 16 pyroptosis-related genes in the high- and low-risk groups.

A single variable lasso technique and Cox regression analysis were used to examine survival-related pyroptosis genes. To create a BC predictive risk model, Lasso regression analysis was used to create 16 pyroptosis genes risk model (Fig. 6A,B). The survival duration of the low-risk group was much longer than that of the high-risk group, according to a Kaplan Meier analysis (Fig. 6C–E). With a median risk score of 1.96 (Fig. 6I–K), the number of deaths in the high-risk group increased dramatically (Fig. 6L–N). In order to assess the risk model’s predictive value in the BC cohort. The risk score ROC curve for 1, 3, and 5-year survival time (Fig. 6F–H) was further examined, indicating that it has high sensitivity and specificity for survival prediction. Meanwhile, the risk model’s expression of 16 genes was assessed (Fig. 6O–Q).

$$\begin{aligned} {\text{Risk score}} = & ({\text{expression of BIRC3}} \times - \,0.{187}) \, + \, ({\text{expression of MCOLN2}} \times - \,0.{285}) \, \\ & + \, ({\text{expression of MGAT1}} \times 0.{657}) \, + \, ({\text{expression of TBPL1}} \times 0.{528}) \, \\ & + ({\text{expression of KIR2DL4}} \times -\, 0.{368}) \, + \, ({\text{expression of HCG4B}} \times -\, 0.{3}0{7}) \, \\ & + \, ({\text{expression of GPA33}} \times - \,0.{291}) \, + \, ({\text{expression of HRH2}} \times 0.{417}) \, \\ & + \, ({\text{expression of OSTF1}} \times 0.{496}) \, + \, ({\text{expression of TYK2}} \times -\, 0.{519}) \, \\ & + ({\text{expression of FOXF1}} \times 0.{374}) \, + \, ({\text{expression of MASTL}} \times 0.{28}0{8}) \, \\ & + \, ({\text{expression of UCP3}} \times -\, 0.{392}) \, + ({\text{expression of SLC1A4}} \times -\, 0.{287}) \, \\ & + \, ({\text{expression of IKBKG}} \times 0.{915}) \, + \, ({\text{expression of RSPH1}} \times - 0.{165}). \\ \end{aligned}$$

### Construct a nomogram of pyroptosis risk score

**Figure 7:**
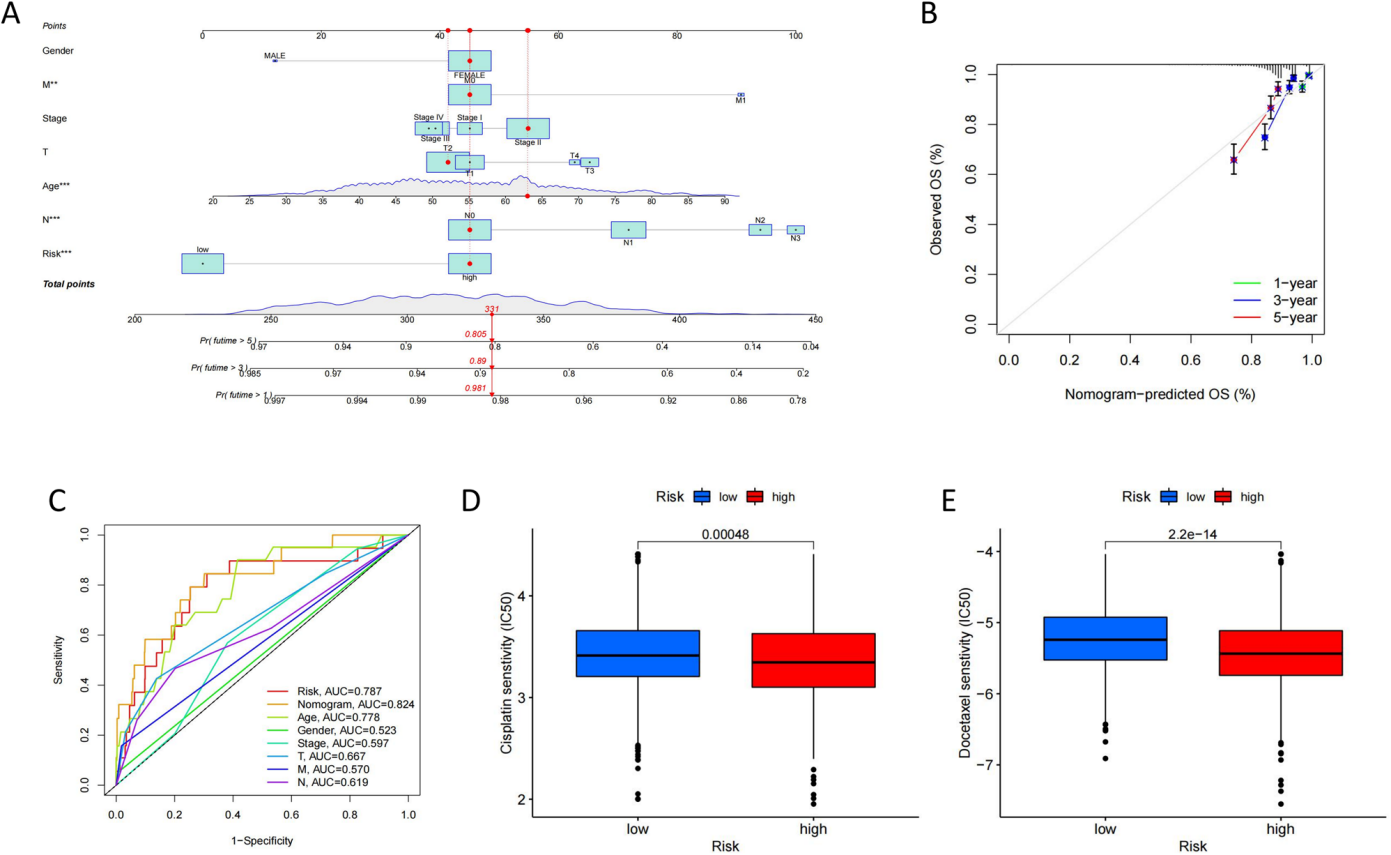
Construction and validation of a nomogram. (**A**) Nomogram for predicting the 1-, 3-, and 5-year OS of BC patients. (**B**) Calibration curves of the nomogram for predicting of 1-, 3-, and 5-year OS in all BC patients. (**C**) ROC curves of risk scores, nomogram and clinicopathological features for all BC patients. (**D,E**) Sensitivity of high and low risk to two chemotherapeutic agents, cisplatin and docetaxel.

A predictive nomogram incorporating risk score and clinicopathological aspects was developed to predict the prognosis of BC patients based on the difference in risk score between distinct clinicopathological variables (Fig. 7A). The calibration curve approximates the diagonal, indicating that in our nomogram, 1, 3, and 5-year OS have a good predictive capacity in our nomogram (Fig. 7B). The risk score and nomogram are effective predictors (Fig. 7C). The low-risk group was shown to be more susceptible to cisplatin and docetaxel. The low-risk group was more sensitive to both Cisplatin and Docetaxel (Fig. 7D,E). We determined through univariate and multivariate Cox regression analysis that the risk score was an independent prognostic factor influencing breast cancer patients (Supplement Fig. S5A,B).

### Correlation between risk score and characteristics of tumor immune microenvironment

**Figure 8:**
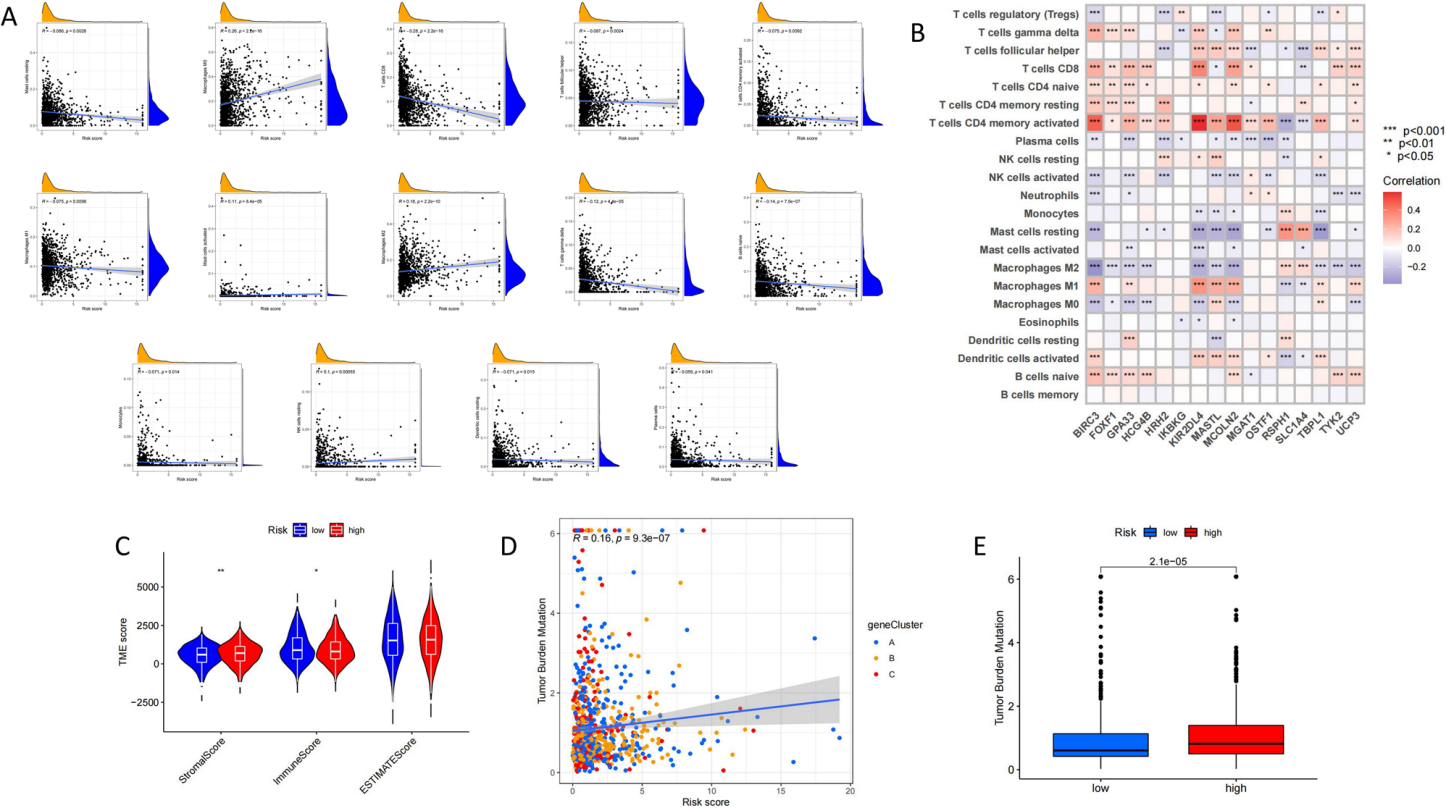
Correlation between risk scores and characteristics of the tumor immune microenvironment. (**A**) Correlation between risk score and immune cell type. (**B**) Correlation between the abundance of immune cells and the 16 genes in the risk model. (**C**) Correlation between risk scores and immune cell and stromal cell scores. (**D**) Correlation between risk score and TMB and genotyping. (**E**) The role of TMB in different risk score groups.

It demonstrates that the prediction model’s risk score is closely linked to immunity. Further analysis revealed that the risk score was positively correlated with M0 macrophages, M2 macrophages, mast cell activation, and NK cell resting content, and negatively correlated with B cells naive, dendritic cells resting, CD4 memory T cell activation, T cells CD8, T cells regulatory (Tregs) and M1 macrophages, implying that immune cell infiltration in the high-risk group was reduced, resulting in a decline of immune function (Fig. 8A). The relationship between 16 genes in the model and the number of immune cells was evaluated. It was discovered that these 16 genes were highly linked to the majority of immune cells (Fig. 8B). The analysis indicated that a low TME score is strongly associated with a high immune score, while a high TME score is closely related to a high matrix score in order to further investigate if the risk score may be utilized as an immune index (Fig. 8C). And in the high-risk group TMB was higher (Fig. 8D), TMB also positively connected with the high-risk score (Fig. 8E), implying that the high-risk group is more likely the failure immunotherapy.

## Discussion

Breast cancer is the major cause of cancer-related fatalities in women (approximately 15% of all cancer-related deaths in women)^1^. In addition to surgery, targeted therapy and chemotherapy are frequently used to control/shrink bigger tumors and lower the risk of recurrence and metastasis^45^. After treatment, most tumors trigger programmed cell death^46^, and associated cell death killing breast cancer cells. Caspase-3 activation by chemotherapeutic medicines causes secondary necrosis/pyroptosis of cancer and normal cells and plays a significant role in cancer chemotherapy^47^. Pyroptosis is the formation of pores on the plasma membrane, leading to the destruction of the cell's permeability barrier and subsequent cell enlargement^48^. Active caspase-3 cleaves GSDME to create the N-terminal fragment of GSDME (GSDME-NT) when apoptosis commences. GSDME-NT will translocate and perforate, causing pyroptosis^49^. CNV is a structural variation that accounts for 4.8% to 9.5% of human genome diversity^50^. Some CNVs in TNBC can indicate poor prognosis and can act as prognostic markers, and they may be connected to lymph node metastasis^51^. Based on the TCGA cohort and GEO dataset, we initially looked at pyroptosis regulating gene mutations in BC. BC demonstrated a unique somatic mutation due to its heterogeneity, according to the findings. The pyroptosis regulatory genes of CNV had varying degrees of deletion and amplification and the mutation rate of somatic cells was as high as 30.93%, showing that pyroptosis regulatory gene mutations play an essential role in breast cancer.

To validate the accuracy of our findings using a single dataset, we carried out further association analysis using multiple other GEO datasets by cluster analyses. These datasets with different molecular features are combined to achieve improved normalization^52^. Association outcomes reflect the differences between single and grouped/cluster dataset analysis. Clustering analysis of each subtype offers concentrated groups and amplifies the molecular typing differences. Clustering analysis also improved the accuracy of tumor subtype classification^53^.

Increasing data suggest that tumor pyroptosis is related to tumor formation and progression. Pyroptosis has been to slow the growth of lung cancer tumors^54^, gastric cancer^55^, and colorectal cancer^56^. Pyroptosis can activate the innate immune system, inhibit the development of tumor cells by changing TME, and even directly kill tumor cells^39^. DEGs screened according to three pyroptosis subtypes were shown to be involved in T cell activation and cytokine interaction in this validation, indicating that breast cancer is intimately linked to inflammation and immunological modulation. Inflammatory bodies and IL-1 have been linked in recent studies showing that they play a vital function in promoting breast cancer growth and metastasis. Tumor growth is linked to an elevated level of IL-1 in the tumor microenvironment in mice mammary tumor models and human breast cancer tissues, which increases the infiltration of myeloid cells like tumor-associated macrophages (TAMs) and myeloid-derived suppressor cells^57^.

This study discovered that there was a clear enrichment of immune cells in Cluster C, as well as enrichment associated with immune activation, implying that localized death may play a role in breast cancer immune regulation. We also developed a pyroptosis score quantitative approach to identify different pyroptosis regulatory gene subtypes and serve as a guide for individual evaluation and treatment choices in this investigation. The immune activation pyroptosis pattern had a higher score and a better prognosis, according to the findings. TMB is one of the newest biomarkers in the field of cancer immunotherapy^58^, TMB is considerably greater in ER-negative BC individuals, particularly in TNBC patients^59^. The pyroptosis score is linked to the number of TMB in this study. The better the prognosis of patients with high TMB, the higher the pyroptosis score, showing that the pyroptosis score can be utilized as an independent prognostic marker.

According to the results of the analysis, the survival prognosis of the high pyroptosis score in our model was better than the low score, it was found that in the pyroptosis model of gastric cancer^60^, melanoma^61^, head and neck squamous cell carcinoma^62^, patients with high scorch death scores also had a good prognosis, and the immunotherapy effect was better than that of the low pyroptosis score group, which was consistent with our research results. However, the prognosis of low scores in low-grade gliomas^63^ indicates that the scorch death score may play an important role in different tumors, which can better predict the TME status of tumors.

It has been found that PD-L1 is significantly more expressed in breast cancer tissues, especially in triple-negative breast cancer than in normal breast tissue, and the safety and efficacy of PD-L1 inhibitor pembrolizumab in triple-negative breast cancer, hormone receptor positive, HER2-negative, local recurrence or metastatic breast cancer are significantly enhanced^64,65^.

In this study, the expression of PD-L1, CTLA4, PDCD1, PDCD1LG2 and HAVCR2 in high focus death score was found to increase, and combined with the results of the literature, it can be shown that immunotherapy in patients with PD-1/PD-L1-positive breast cancer has significant therapeutic advantages and clinical efficacy. Similar therapeutic effects were also found in lung cancer^66^ and melanoma^67^, consistent with our findings. Clinical trials have shown that tumor cells with higher levels of TMB are more easily recognized by the immune system and therefore have a stronger immune response to immune checkpoint inhibitors. If the tumor mutation burden is greater, there may be a good response to immunotherapy drugs (PD-1/PD-L1 inhibitors) Nivolumab, Pembrolizumab and Atezolizumab^68,69^.

Pyroptosis enhances immune activation and function, resulting in tumor clearance. Furthermore, tumor cells can activate pyroptosis in a variety of ways, and some immune cells can directly generate it, implying that pyroptosis is implicated in the positive feedback control of anti-tumor immunity^70^. In BALB/c mice treated with NP-Gsdma3 and Phe-BF3, the number of CD4+, CD8+, natural killer (NK), and M1 macrophages increased. Monocytes, neutrophils, myeloid-derived suppressor cells, and M2 macrophages all decreased, implying that pyroptosis may play a role in tumor immune control^71^. GSDMB-mediated pyroptosis can function downstream of GZMA, and cytotoxic lymphocytes can transmit GZMA to GSDMB-expressing cancer cells, enhancing antitumor immunity^72^.

This study was a retrospective analysis using information from the database. Selective bias could skew the results; therefore, more data from BC patients undergoing immunotherapy is needed to confirm the study's conclusions. Clinical data such as surgery, neoadjuvant chemotherapy, radiation, and chemotherapy are not studied, which could alter the immune response and pyroptosis prognosis.

## Conclusion

In this investigation, we genotyped BC samples using 40 pyroptosis genes to assess the effect on tumour immune matrix milieu, clinicopathological characteristics, and prognosis in BC patients. The therapeutic benefits of different subtypes and immunotherapy were investigated using a pyroptosis prognostic model. This study adds to our knowledge of the regulatory role of pyroptosis genes in BC. Findings also provide a valuable reference for guiding personalised immunotherapy and BC prognosis.

## Supplementary Information

Supplementary Legends.

Supplementary Figure S1.

Supplementary Figure S2.

Supplementary Figure S3.

Supplementary Figure S4.

Supplementary Figure S5.

Supplementary Table S1.

## Data Availability

The datasets used and/or analyzed during the current study available from the corresponding author on reasonable request.

## Abbreviations

BC: Breast cancer
PRGs: Pyroptosis-related genes
TMB: Tumor mutation burden
UCSC: The University of California, Santa Cruz
TCGA: The Cancer Genome Atlas
GEO: Gene Expression Omnibus
CNV: Copy number variation
TME: Tumor microenvironment
GSEA: Gene Set Enrichment Analysis
HMGB1: High mobility group box 1
LPS: Lipopolysaccharide
TNBC: Triple negative breast cancer
TILs: Tumor-infiltrating lymphocytes
CAR-T: Chimeric Antigen Receptor T-Cell Immunotherapy
ICIs: Immunosuppressive point inhibitors
GSDME: Gasdermin E
DEGs: Differentially expressed genes
PCA: Principal component analysis
MAF: Mutation annotation format
CDF: Cumulative distribution function
AUC: Area under the curve
ssGSEA: Single sample gene set enrichment analysis
TICs: Tumor-infiltrating immune cells
GO: Gene Ontology
KEGG: Kyoto Encyclopedia of Genes and Genomes
PD-L1: Programmed cell death-Ligand 1
CTLA4: Cytotoxic T-lymphocyte-associated protein 4
OS: Overall survival
Treg cells: Regulatory T cells
ROC: Receiver Operating Characteristic Curve
IC50: Half maximal inhibitory concentration
TAMs: Tumor-associated macrophages
