# Supplementary material for: Identification of pyroptosis related subtypes and tumor microenvironment infiltration characteristics in breast cancer
Source: Sci Rep. 2022 Jun 23;12:10640. doi: 10.1038/s41598-022-14897-1 (PMC9226023; doi:10.1038/s41598-022-14897-1)
Supplement: Supplementary file 1 — Supplementary Legends. [file 41598_2022_14897_MOESM1_ESM.doc]

**Figure S1. Survival analysis revealed that 29 PRGs were closely associated with prognosis.** Patients with high expression of AIM2, CASP1, CASP4, CASP8, CHMP2A, CHMP4B, CHMP6, CHMP7, ELANE, GSDMD, GZMA, GZMB, IL1A, IL1B, IL18, IRF1, IRF2, NAIP, NLRC1, TP63 and ZBP1 had a better prognosis, while patients with low expression of these genes had a better prognosis. Patients with low expression of BAK1, CHMP2B, CHMP4C, CYCS, DHX9, GSDMB, GSDMC and NLRC4 had a better prognosis.

**Figure S2. Validation of PRGs subtypes.** (A) Consensus matrix heat map defining the three clusters (k = 3) and their associated regions. (B) Survival analysis of the three subtypes versus OS. (C) PCA analysis showing significant differences in transcriptomes between the three subtypes. (D) Differences in the three subtypes in relation to clinicopathological features and expression levels of PRGs.

**Figure S3**. **Clinical significance of the pyroptosis score.** (A) Analysis of the prognostic predictive power of the pyroptosis score in different clinical subtypes. (B) Relationship between pyroptosis score and characteristics of different clinical subtypes.

**Figure S4. The ROC analysis at 1,3,5 years of overall survival for our pyroptosis -16 gene, Wu-17 genes, Yu-15 genes and Xu 3 genes signatures in the all TCGA cohort.** (A-D) AUC values comparison with Wu-17 genes, Yu-15 genes and Xu 3 genes signatures, (E-H) OS comparison with Wu-17 genes, Yu-15 genes and Xu 3 genes signatures, (I-G) C-index and RMS index in four models.

**Figure S5. Multivariate Cox regression analysis that the risk score was an independent prognostic factor influencing breast cancer patients.** A: Univariate Cox, B: Multivariate Cox.
