# Supplementary figures and images for: Identification of pyroptosis related subtypes and tumor microenvironment infiltration characteristics in breast cancer
Source: Sci Rep. 2022 Jun 23;12:10640. doi: 10.1038/s41598-022-14897-1 (PMC9226023; doi:10.1038/s41598-022-14897-1)

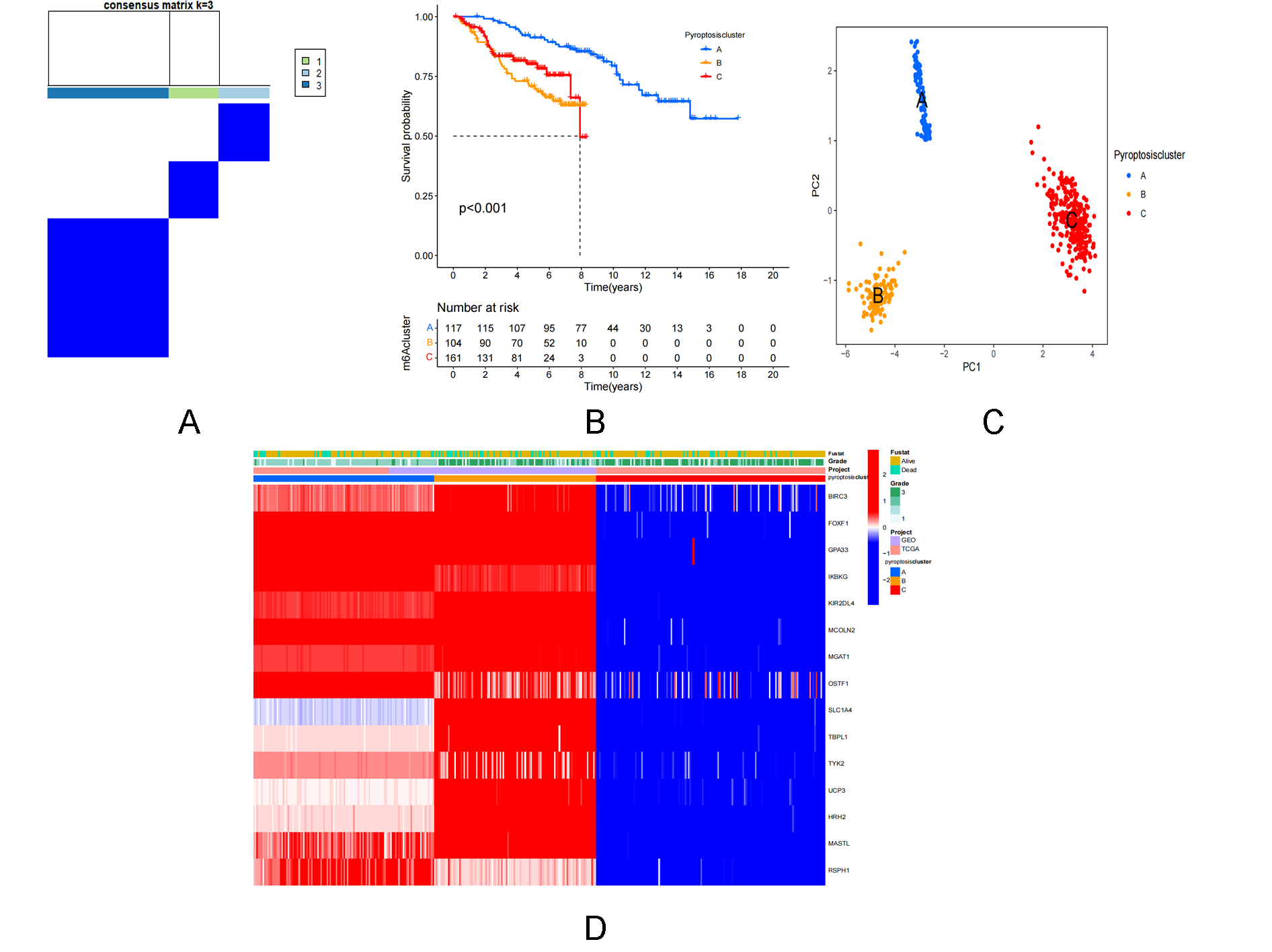

Supplement: Supplementary file 3 — Supplementary Figure S2. [file 41598_2022_14897_MOESM3_ESM.tif]

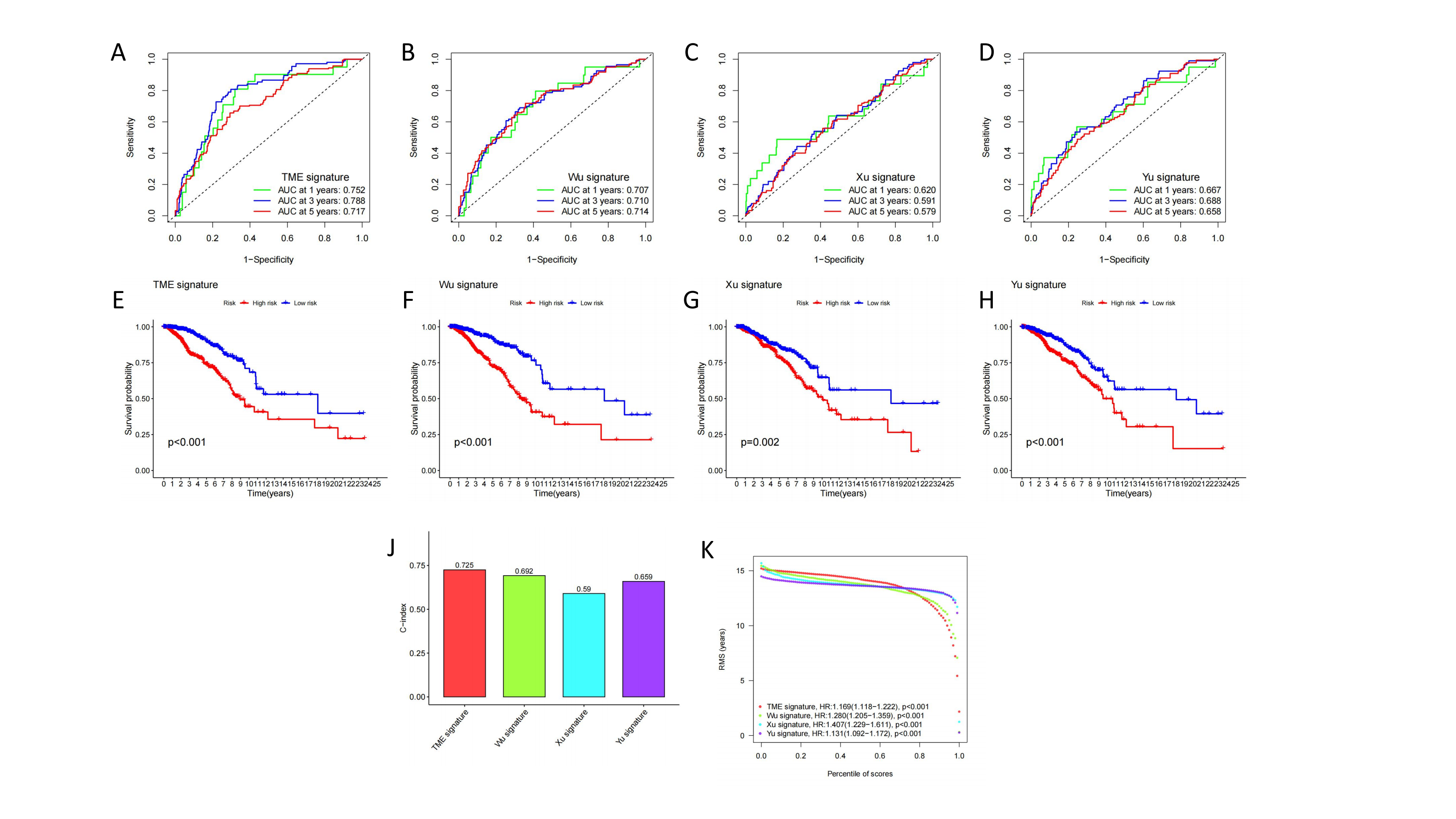

Supplement: Supplementary file 5 — Supplementary Figure S4. [file 41598_2022_14897_MOESM5_ESM.tif]

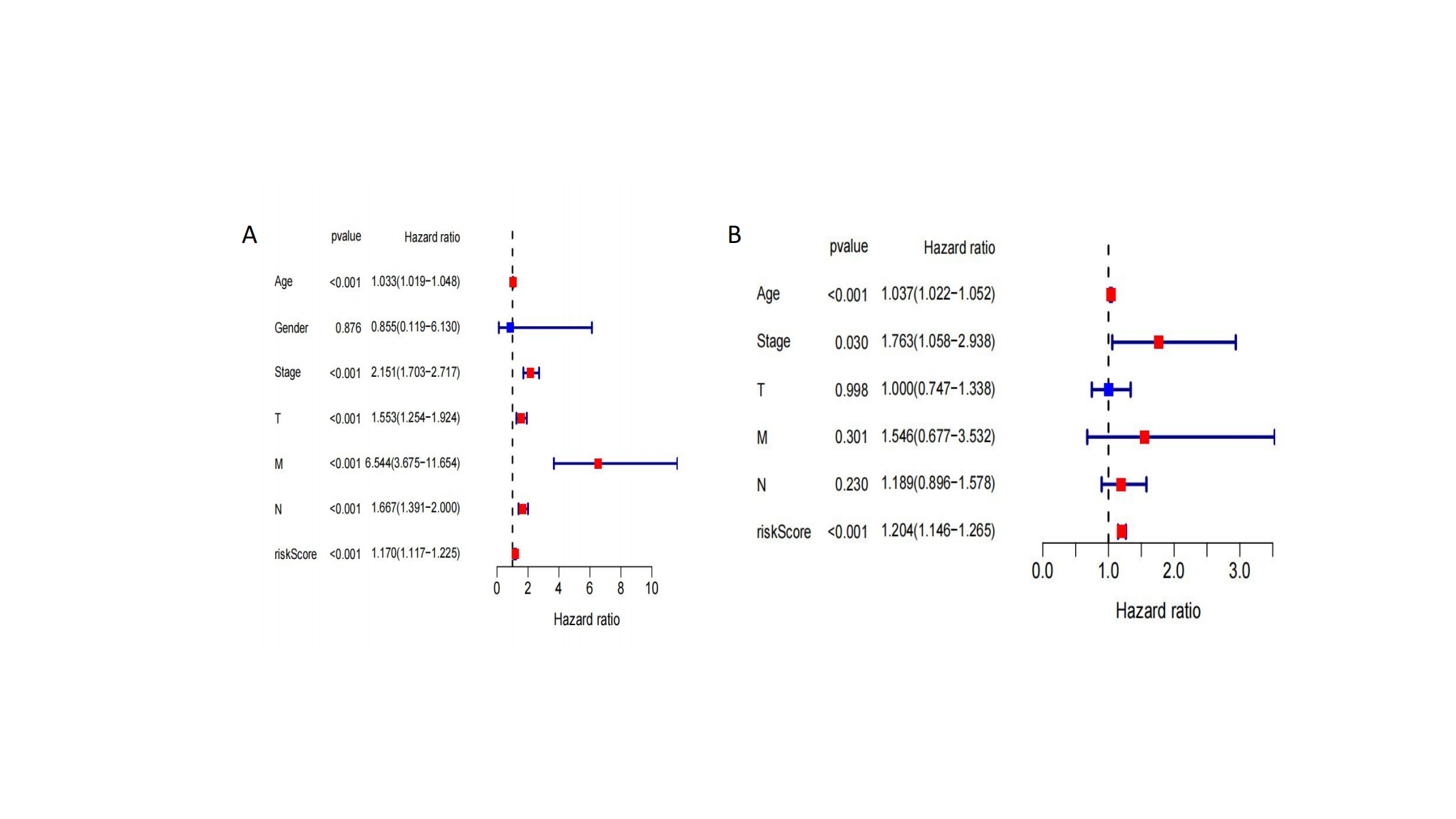

Supplement: Supplementary file 6 — Supplementary Figure S5. [file 41598_2022_14897_MOESM6_ESM.tif]
