# Supplementary material for: Identification of pyroptosis related subtypes and tumor microenvironment infiltration characteristics in breast cancer
Source: Sci Rep. 2022 Jun 23;12:10640. doi: 10.1038/s41598-022-14897-1 (PMC9226023; doi:10.1038/s41598-022-14897-1)
Supplement: Supplementary file 7 — Supplementary Table S1. [file 41598_2022_14897_MOESM7_ESM.doc]

Supplemental Table S1 Multivariate Cox analysis selection genes.

| Gene | coef |
| --- | --- |
| BIRC3 | -0.187707368 |
| MCOLN2 | -0.285735393 |
| MGAT1 | 0.657257622 |
| TBPL1 | 0.528228257 |
| KIR2DL4 | -0.368787631 |
| HCG4B | -0.307577892 |
| GPA33 | -0.290890404 |
| HRH2 | 0.41798987 |
| OSTF1 | 0.496339579 |
| TYK2 | -0.519797631 |
| FOXF1 | 0.374123758 |
| MASTL | 0.280860775 |
| UCP3 | -0.392394759 |
| SLC1A4 | -0.287808187 |
| IKBKG | 0.915481444 |
| RSPH1 | -0.164723822 |
